# Advances and potential new developments in imaging techniques for posterior uveitis Part 2: invasive imaging methods

**DOI:** 10.1038/s41433-020-1072-0

**Published:** 2020-08-10

**Authors:** Carl P. Herbort, Ilknur Tugal-Tutkun, Alessandro Mantovani, Piergiorgio Neri, Moncef Khairallah, Ioannis Papasavvas

**Affiliations:** 1 Retinal and Inflammatory Eye Diseases, Centre for Ophthalmic Specialized Care (COS), Clinic Montchoisi Teaching Centre, Lausanne, Switzerland; 2 Department of Ophthalmology, Istanbul Faculty of Medicine, Istanbul University, Istanbul, Turkey; 3 Ophthalmology Unit, Ospedale Valduce, Como, Italy; 4 The Eye Institute, Cleveland Clinic Abu Dhabi, Abu Dhabi, United Arab Emirates; 5 Cleveland Clinic Lerner College of Medicine, Case Western Reserve University, Cleveland, OH USA; 6 Department of Ophthalmology, Fattouma Bourguiba University Hospital, Faculty of Medicine, University of Monastir, Monastir, Tunisia

**Keywords:** Uveal diseases, Medical imaging

## Abstract

The aim of this review was to identify the imaging methods at our disposal to optimally manage posterior uveitis at the present time. The focus was put on methods that have become available since the 1990s, some 30 years after fluorescein angiography had revolutionised imaging of posterior uveitis in particular imaging of the retinal vascular structures in the 1960s. We have focussed our review on precise imaging methods that have been standardised and validated and can be used universally thanks to commercially produced and available instruments for the diagnosis and follow-up of posterior uveitis. The second part of this imaging review will deal with invasive imaging methods and in particular ocular angiography.

## 摘要

本文旨在确认当前可行的影像学方法, 以优化后葡萄膜炎的治疗。聚焦于自20世纪90年代以来可行的方法, 30年前荧光素血管造影术对后葡萄膜炎的成像进行了变革, 尤其是20世纪60年代针对视网膜血管结构的成像。本综述着重阐述已标准化及被验证的精确的成像方法, 商业化生产它们得以普遍使用, 且可用于后葡萄膜炎的诊断及随访。本文的第二部分将讨论侵入性影像学方法, 尤其是眼血管造影术。

## Introduction

As indicated in the first part of the review on imaging of posterior uveitis, the huge progress made in imaging techniques in the last two to three decades transformed the appraisal of posterior uveitis into an exact clinical science. The problem however lies in the reluctance to embrace such a new precise and comprehensive approach, perpetuating traditional mostly obsolete approaches promoted by “consensus” conferences and adopted by official health agencies, failing to recommend precise and objective investigations, so not only hampering the diagnosis and management of posterior uveitis worldwide but also hampering the proper design of clinical trials. It is hard to believe that the standard outcome measure for studies on posterior uveitis recommended by the FDA (Food and Drug Administration of the USA) is vitreous haze based on a system proposed more than 40 years ago [1] and taken over without change by the standardisation of uveitis nomenclature group [2]. It is obvious to most of us that a substantial proportion of posterior uveitis entities take place and develop in the choroid with no or minimal vitreous haze making this parameter unsuitable as an outcome measure in most studies. The reluctance to use invasive methods exposed in this second part on imaging methods is especially pronounced despite the fact that choroidal inflammatory diseases are most precisely investigated by indocyanine green angiography (ICGA). So, consensus conferences held in ICGA refractory geographical areas were at the origin of inappropriate disease definitions.

HLA-A29 birdshot retinochoroiditis (BRC) is one example of an unsuitable approach because of the publication of inadequate diagnostic criteria [3]. Clinicians were misled in the diagnosis and management of BRC because the report on the workshop on diagnostic criteria of BRC neglected the crucial importance of HLA-A29 antigen testing and completely ignored ICGA without which early diagnosis and precise follow-up is not possible [4, 5].

Another example is Vogt–Koyanagi–Harada (VKH) disease. Again a consensus workshop failed to include in their report on “the revised diagnostic criteria of VKH” the single most important imaging investigational method, ICGA, for a disease originating exclusively and involving principally the choroid [6]. It is very well established that VKH cannot be precisely followed without the use of investigational imaging of the choroidal compartment [7].

The approach of these diseases was biased because the most adequate imaging methods for choroiditis were not taken into account. Today we dispose of an array of precise imaging methods for posterior uveitis, the problem and the enigma being that so many centres often use them with reluctance [8].

The aim of this review will concentrate on the progress made in the precision with which posterior uveitis can be investigated nowadays thanks to new or revisited techniques that have become available for routine use, such as laser flare photometry, optical coherence tomography (OCT) and fundus autofluorescence, presented in the first part of the review on imaging in posterior uveitis, and fluorescein angiography (FA), ICGA and dual FA/ICGA scoring presented in this second part of the review [9, 10].

## Fluorescein angiography (FA)

FA has been practised since more than 50 years. When it was developed by Novotny and Alvis in the early 1960s it represented a major progress in imaging of the posterior segment [11]. Since then it was possible to analyse blood flow in retinal vessels and determine whether there was abnormal circulation, abnormal vessels or inflammation of vessels. For inflammatory diseases it became an important investigative procedure because it could give additional information on inflammatory events that were not seen otherwise. FA was giving more dynamic information and was adding an element of grading of inflammatory activity thanks to a precise dual FA/ICGA scoring system [12, 13]. FA was shown to be very sensitive to explore inflammatory lesions of the superficial structures of the fundus such as the retina and optic disc, giving also information on the damage to the retinal pigment epithelium (RPE) and on the choriocapillaris in the first 60 s of the angiographic sequence. The drawback of FA was, however, that it could not give information on the choroid, the layer under the retina because there was a screen, the RPE, that did not allow to see the circulation in choroidal vessels. Exploration of the deeper structures was therefore limited by the fact that the RPE was blocking visible light that was entering the eye and also blocked a great part of fluorescing light coming from the choroid.

We will not go into the details of this method used and extensively described in innumerable, articles and books during its more than 50 years of use [14–16].

Although the periphery of the retina was always reachable by taking panorama pictures, recently developed wide-field angiographs have facilitated the access to the periphery of retina and have again attracted attention to the angiographic analysis of these areas [17].

We will briefly review its main uses for inflammatory diseases. Because of the physical–chemical properties of the fluorescein sodium molecule (low molecular weight), there is easy extrusion of fluorescein out of inflamed retinal vessels into tissues from which it is however also quickly washed out.

The main structures explored by FA and the information asked to FA are exposed hereunder.

**Fig. 1:**
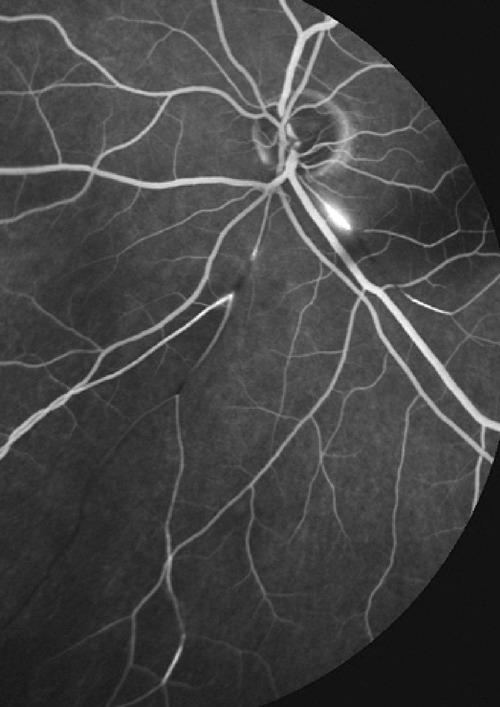
FA showing abrupt and segmental interruption of arteries characteristic of Susac syndrome.

**Fig. 2:**
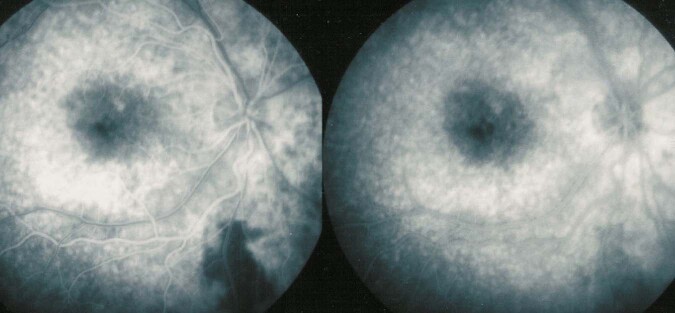
Acute stage of Birdshot HLA-A29 retinochoroiditis. Diffuse exudation from retinal capillaries in such a proportion that the main veins never become hyperfluorescent because there is not enough dye to mark the large veins of the arcades.

**Fig. 3:**
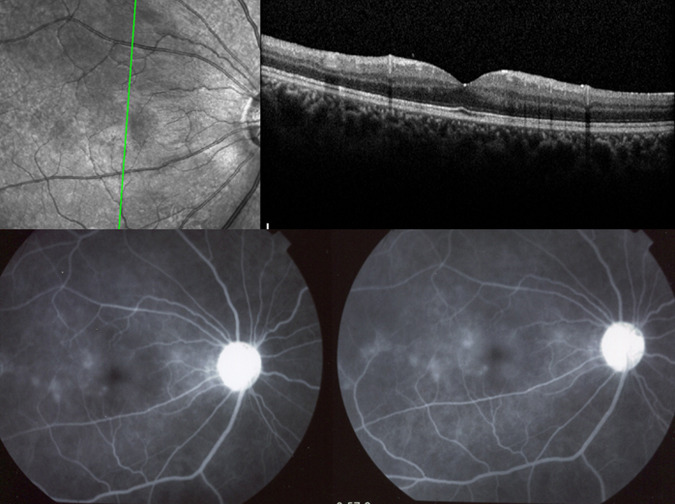
Inflammatory macular oedema in a case of Behçet uveitis. Oedema is clearly outlined on FA (two bottom pictures) but barely shown on the SD-OCT section (top).

1. Optic disc: the degree of papillitis and the presence of subclinical disc inflammation, as well as the detection of pre-papillary neovascularisation are advantageously analysed by the type and degree of FA hyperfluorescence.

2. Retinal vessels: are best analysed by FA that gives (1), very sensitive information on retinal vasculitis even when it is minimal, (2) displays occlusive vasculopathy of retinal arteries or arterioles (Fig. 1) and (3) detects preretinal neovascularisation on disc (NVD) or elsewhere (NVE).

3. The retina: FA is the investigation of choice to image retinal foci which are hypofluorescent in the early angiographic phase, becoming hyperfluorescent in the late phase. In case of widespread leakage of small vessels such as in the active phase of HLA-A29 BRC the whole retina becomes diffusely hyperfluorescent indicating massive tissue staining (Fig. 2).

At the level of the macula, macular ischaemia is very well detected by FA showing increased size of the central avascular zone, such as in Behcet uveitis. FA is also more sensitive than OCT to account for inflammatory macular oedema (Fig. 3).

**Fig. 4:**
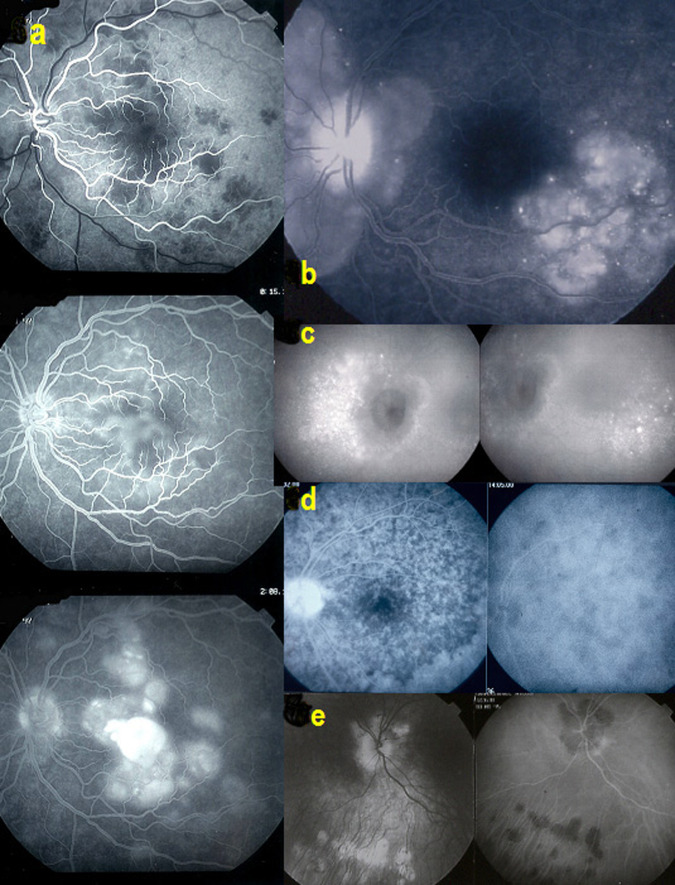
FA signs: intraretinal pooling, exudative retinal detachment and atrophy. **a** FA shows choriocapillaris non-perfusion (top) in a patient diagnosed as APMPPE; gradually there is intraretinal and subretinal pooling (**a** two bottom pictures) due to extrusion of fluorescein coming from the inner retinal capillaries in response to outer retinal ischaemia due to choricapillaris non-perfusion. **b** FA exudative retinal detachment (ERD) with hyperfluorescent pinpoints where choroidal leakage is occurring in a case of VKH disease. **c** Same fundus frame on ICGA showing hyperfluorescence of ERD as well as hyperfluorescent pinpoints. **d** FA shows papillitis and mottled RPE with well defined limits between diseased and healthy RPE (high water marks) in a case of VKH (left picture). The right picture is an ICG frame of the same area showing numerous persisting hypofluorescent dark dots (HDDs) indicating active disease. **e** Chorioretinal atrophy, late hyperfluorescent on FA and hypofluorescent on ICGA.

4. Subretinal space: FA is giving information on subretinal fluid accumulation. Such subretinal pooling of fluorescein can be of retinal origin as in choriocapillaritis. Inflammatory choriocapillaris non-perfusion produces ischaemia in the outer retina which in turn produces reactional leakage from internal retinal vessels. One example of this process is illustrated by severe cases of acute posterior multifocal placoid pigment epitheliopathy (APMPPE) (Fig. 4a—left column of 3 pictures). Another mechanism of subretinal pooling can be produced by massive choroidal inflammation such as in VKH disease and the fluid in this case is of choroidal origin and produces exudative retinal detachments. FA also shows hyperfluorescent pinpoints marking the leaking points of the outer blood-retinal barrier through which fluid is escaping towards the subretinal space (Fig. 4b, c).

5. The retinal pigment epithelium (RPE): lesions are well highlighted by FA. Loss of RPE will appear as hyperfluorescent areas as through such “window-defects” the choroidal fluorescein will become visible. In VKH, exudative retinal detachments damage the RPE producing loss of RPE cells on one side and pigment clumps on the other hand, (masking even more the choroidal fluorescence), which will produce an irregular hyperfluorescent (window-defect) and hypofluorescent (masking effect) giving a mottled aspect of the RPE (Fig. 4d).

6. Choriocapillaris: in the early FA angiographic phase (60″), the amount of fluorescein in the choriocapillaris is such that choriocapillaris perfusion inhomogeneities can be detected which occur in primary choriocapilaritis entities such as APMPPE. (Fig. 4a, top) In subsequent angiographic phases choroidal fluorescence is hidden by the RPE unless there is loss of RPE (window-defect).

7. Chorioretinal atrophy: total chorioretinal atrophy is hyperfluorescent on FA, as the small fluorescein sodium molecule is staining the bare sclera, whereas, on ICGA full-thickness atrophy is hypofluorescent as the large protein-bound ICG molecular complex is not diffusing to the non-perfused atrophic areas (Fig. 4e).

8. Choroidal inflammatory neovascularisation (CNV): can be detected and analysed by FA and represents a further clinicopathological situation usefully investigated by FA.

9. Subretinal fibrosis: as can be found in chronic or end-stage VKH disease or in idiopathic multifocal choroiditis causes FA tissue staining, delineating the fibrosis [18].

## Indocyanine green angiography (ICGA)

### Introduction

**Fig. 5:**
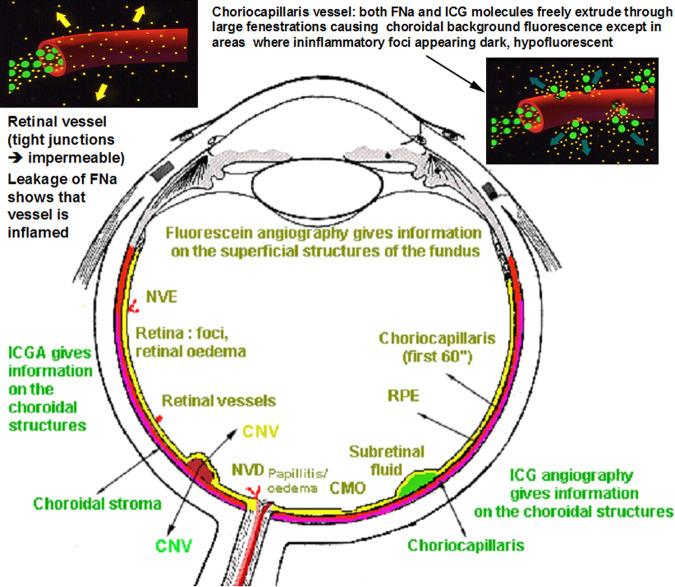
Complementary information from fluorescein fundus angiography (FA) and from indocyanine green angiography (ICGA). FA draws its advantages from the small size of the fluorescein molecule (picture top left) and gives information on all superficial structures of the fundus including preretinal and intraretinal haemorrhages, optic disc, retinal vessels, neovessels, retina, macula, subretinal space, RPE and chorioretinal atrophy, choriocapillaris and choroidal neovessels. ICGA draws its advantage from the infrared fluorescence of the ICG molecule and from its macromolecular behaviour. ICG extrudes from the largely fenestrated choriocapillaris (picture top right), impregnates and is stuck in the choroidal stroma, so lowlighting inflammatory foci seen in dark because diffusion of ICG is impaired. CMO cystoid macular oedema, CNV choroidal neovessels, NVD neovessels disc, NVE neovessels elsewhere, RPE retinal pigment epithelium.

Ocular angiography comprising (Fig. 5) FA and ICGA is still a determining investigation performed for uveitis and is an essential support of the clinical examination in uveitis of the posterior segment. Angiography may be undertaken to better identify elements already detected through the clinical examination or thanks to investigational modalities such as OCT or else. Another motivation to perform an angiography is to better evaluate the degree of inflammation of the fundus. Thirdly, angiography allows to determine a good baseline inventory of inflammatory involvement that will be useful for follow-up purposes. In follow-up situations, angiography is usually performed to monitor disease intensity and impact of therapy.

Because fluorescein sodium fluoresces in the wavelengths of the visible light, mainly gives information on the superficial structures of the fundus as indicated in the previous chapter and thus, often only emphasises signs the clinician has already identified, as, chronologically, OCT is usually already available when FA is performed.

At the beginning of the 1990-decade ICGA was developed and complemented FA. The dye used, indocyanine green (ICG) has the particularity to fluoresce in the infrared wavelengths and therefore gives images of the choroid to which there was poor imaging access before ICGA. In contrast to FA, ICGA often gives additional information, undetected by clinical examination or FA or OCT. In consequence for a thorough assessment of inflammatory involvement in posterior uveitis, ICGA in most instances appears as unavoidable, as it gives information that would otherwise be lost. It is not rare that ICGA shows occult choroidal lesions and therefore has often a diagnostic value, which is rarely the case for FA. Therefore, in most cases where angiographic work-up is deemed necessary and choroidal involvement cannot be excluded a priori, dual FA and ICGA should be undertaken.

### Indocyanine green angiography (ICGA): principles

**Fig. 6:**
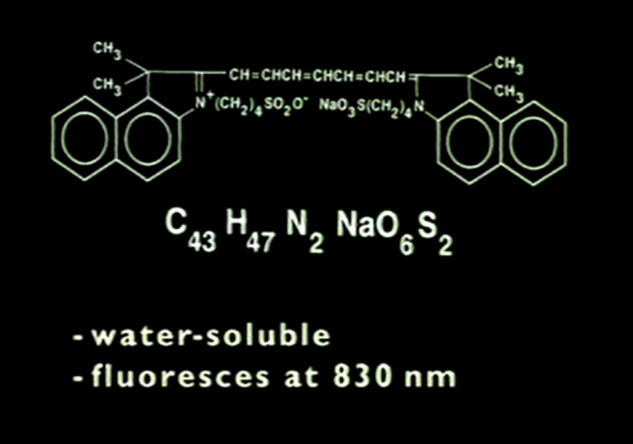
ICG angiographic principles. The ICG molecule (775 daltons) (Fig. 6) is 98% protein bound forming a large macromolecular ICG-protein complex (>58,000 daltons) (Fig. 7) that extrudes freely through the large fenestrations of the choriocapillaris (Fig. 8) progressively impregnating the choroidal stroma (Fig. 9). This determines the normal ICGA pattern (Fig. 10a–c).

**Fig. 7:**
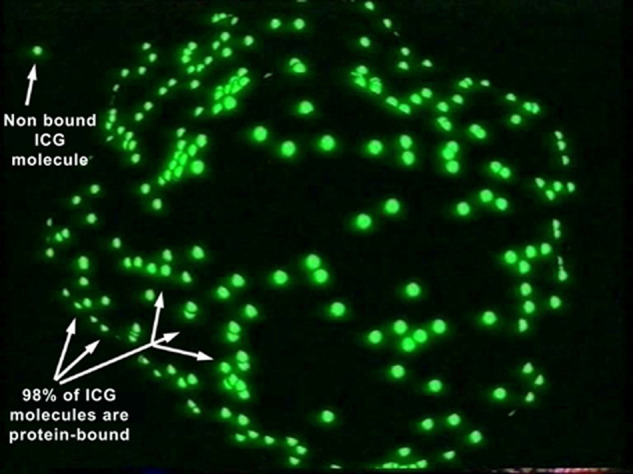
ICG angiographic principles. The ICG molecule (775 daltons) (Fig. 6) is 98% protein bound forming a large macromolecular ICG-protein complex (>58,000 daltons) (Fig. 7) that extrudes freely through the large fenestrations of the choriocapillaris (Fig. 8) progressively impregnating the choroidal stroma (Fig. 9). This determines the normal ICGA pattern (Fig. 10a–c).

**Fig. 8:**
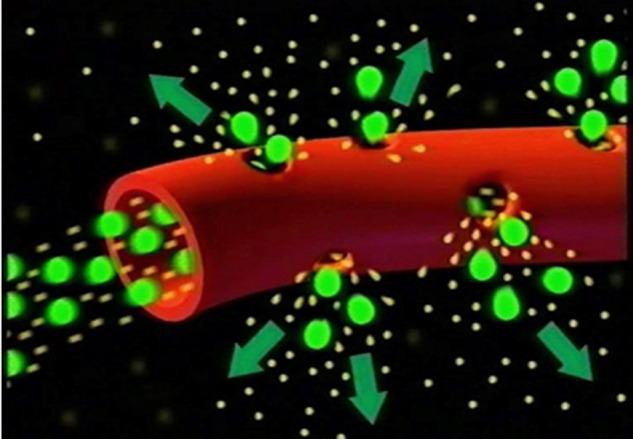
ICG angiographic principles. The ICG molecule (775 daltons) (Fig. 6) is 98% protein bound forming a large macromolecular ICG-protein complex (>58,000 daltons) (Fig. 7) that extrudes freely through the large fenestrations of the choriocapillaris (Fig. 8) progressively impregnating the choroidal stroma (Fig. 9). This determines the normal ICGA pattern (Fig. 10a–c).

**Fig. 9:**
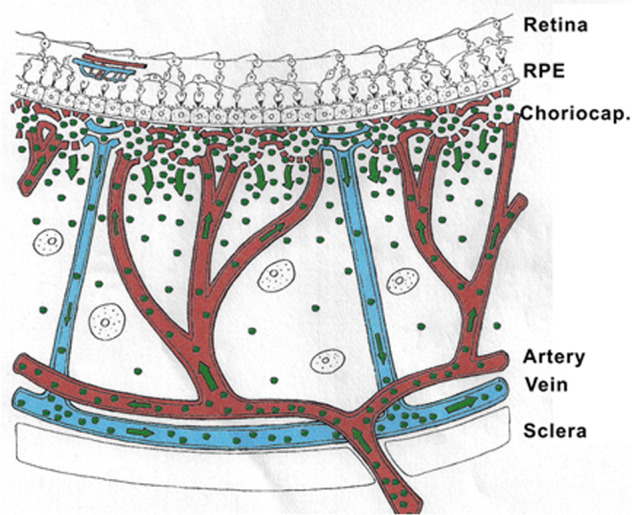
ICG angiographic principles. The ICG molecule (775 daltons) (Fig. 6) is 98% protein bound forming a large macromolecular ICG-protein complex (>58,000 daltons) (Fig. 7) that extrudes freely through the large fenestrations of the choriocapillaris (Fig. 8 progressively impregnating the choroidal stroma (Fig. 9). This determines the normal ICGA pattern (Fig. 10a–c).

ICG fluoresces at 830 nm and therefore is giving access to the choroidal vascular structures through the RPE. The molecular weight difference between ICG (775 daltons) (Fig. 6) and fluorescein (354 daltons) molecules does not account for the specific angiogram pattern obtained with ICG as compared with fluorescein. Beside the different wavelength at which ICG fluoresces, the crucial difference between these two fluorescing molecules is that ICG is nearly completely protein bound and predominantly so to large sized proteins (lipoproteins) forming a macromolecular complex [19] (Fig. 7). Fluorescein leaks readily from slightly inflamed retinal vessels with minor damage and readily impregnates tissues, whereas only major damage to retinal vessels allows ICG to leak. In the choroid however the ICG-protein molecular complex leaks unimpaired but slowly from the fenestrated choriocapillaris (Figs. 8, 9). During recirculation more and more ICG is entrapped in the choroidal tissue as the ICG-protein complex is only slowly reabsorbed into the circulation. Gradual impregnation of the choroid occurs with time causing intermediate and late choroidal background fluorescence that can last for up to 4–5 days. This choroidal impregnation by ICG fluorescence is disturbed by choroidal inflammatory lesions. It is this alteration of the slow choroidal impregnation process that is the main parameter studied in ICGA performed for posterior uveitis.

#### ICGA features in a normal eye

**Fig. 10:**
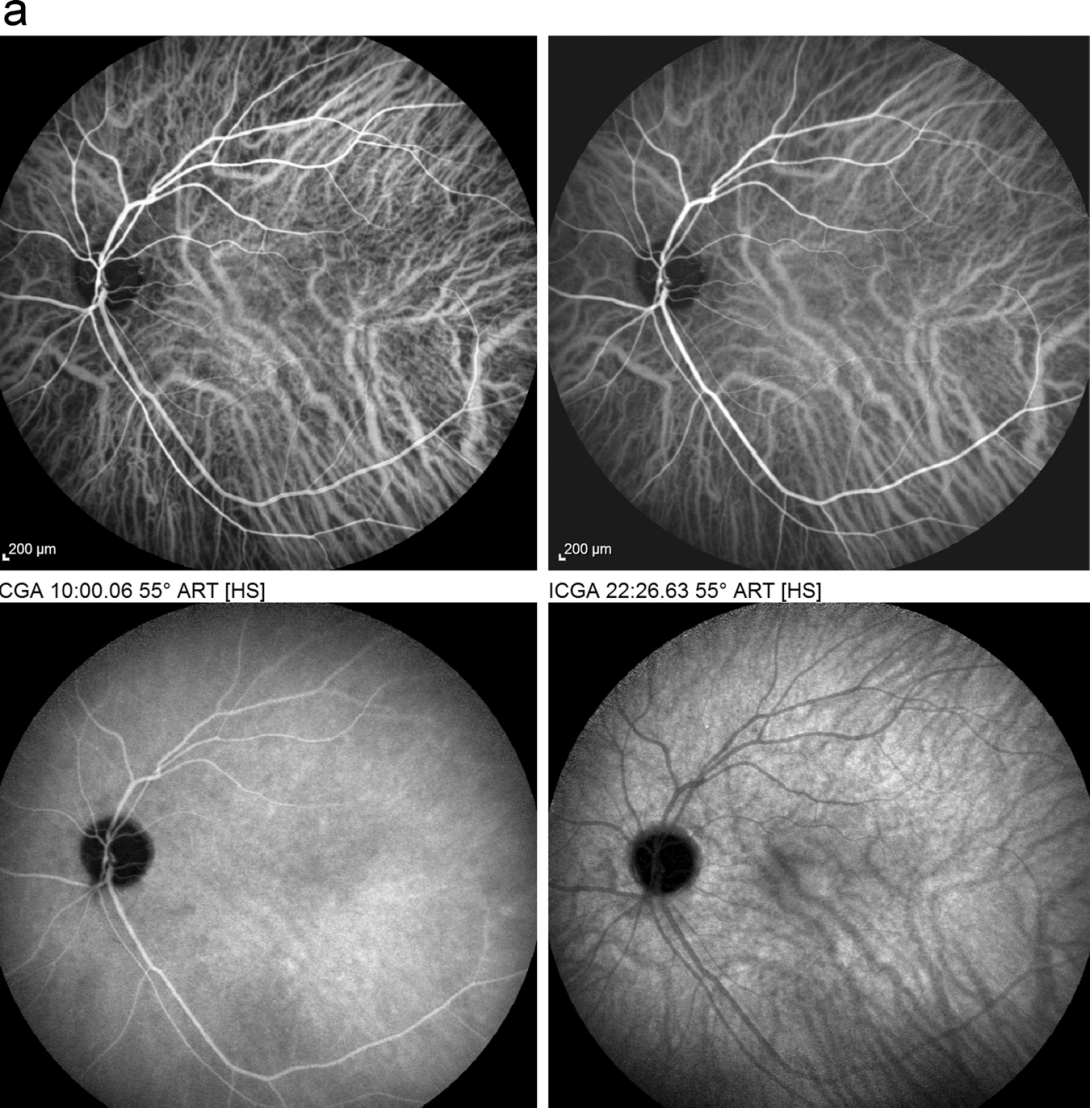

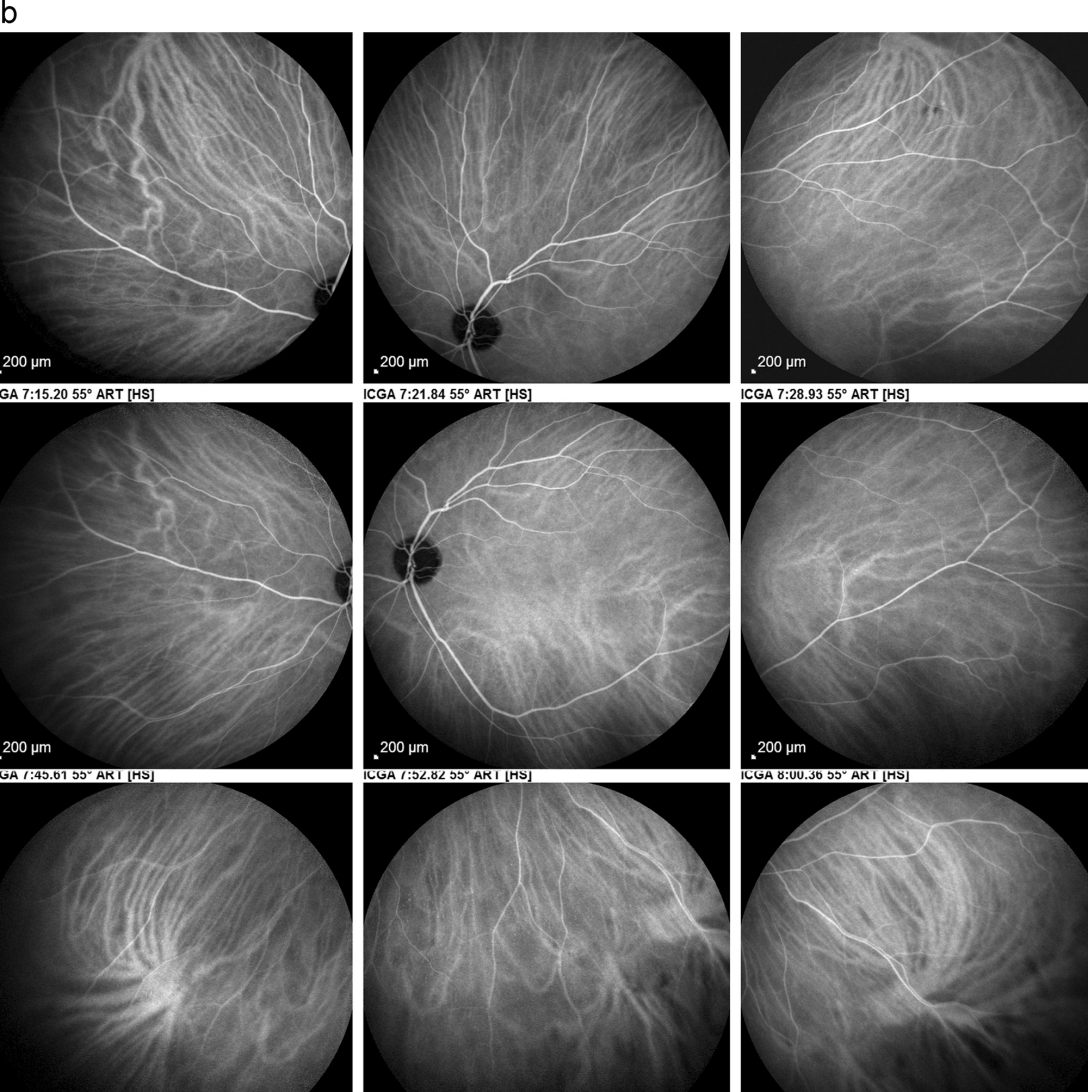

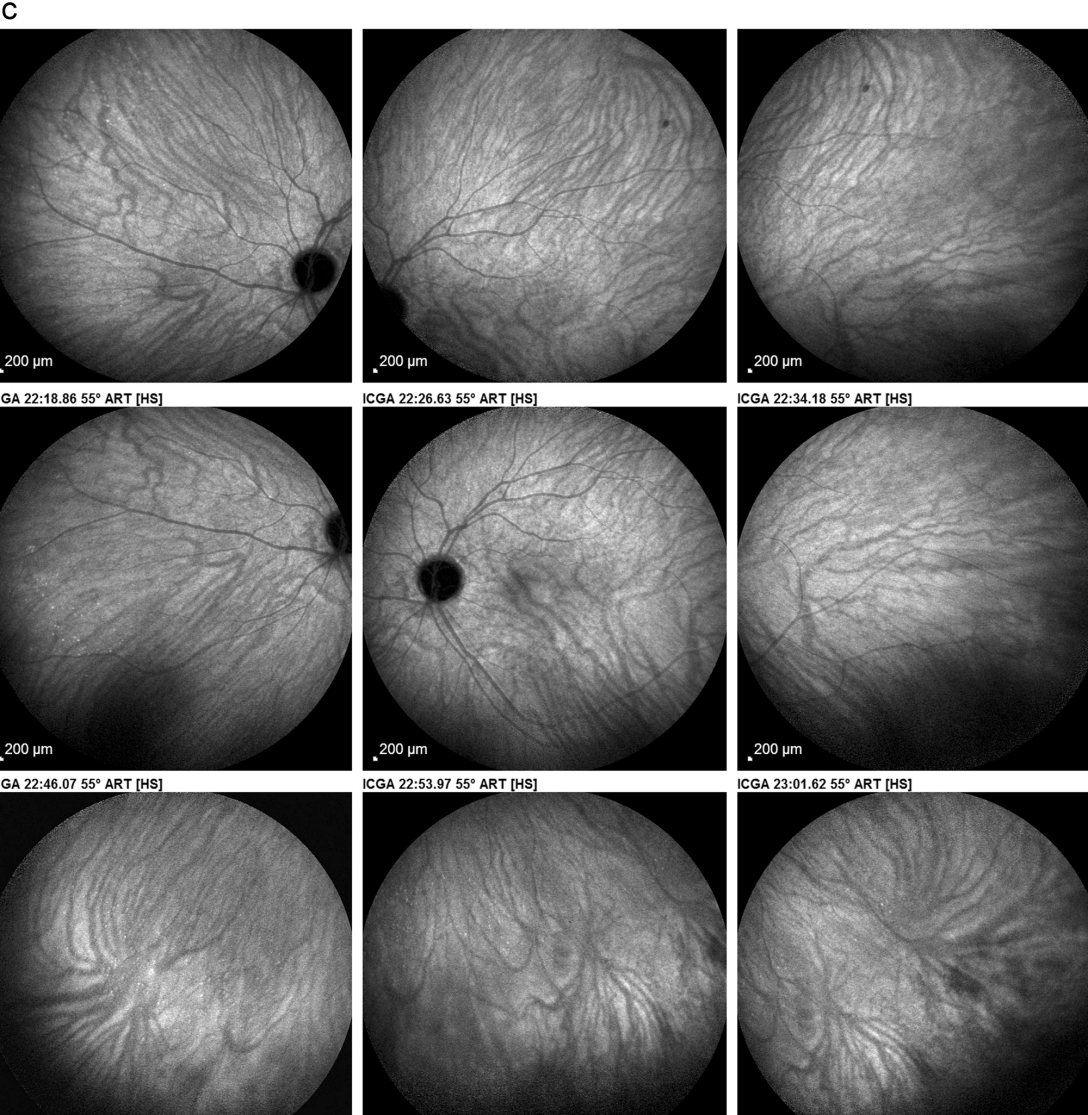
**a** *Normal ICG angiogram*: early phase ICGA angiogram up to 2 min. Showing early choroidal circulation fluorescence positive as well as retinal circulation (top two frames). Two bottom frames show choroidal circulation in negative (dark), as there is no more ICG dye in the circulation but the choroidal vessels come out against the stromal background fluorescence; in the intermediate phase dye is still seen in the retinal vessels (bottom left picture). **b** *Normal ICG angiogram:* intermediate phase showing the superposed retinal vessels and choiroidal vessels both still filled with ICG. **c** *Normal ICG angiogram:* late phase (about 22 ± 4 min) angiogram showing choroidal vessels in a negative pattern detected in dark against the physiological hyperfluorescence background of the choroidal stroma.

ICGA is the method of choice to investigate and follow inflammatory lesions of the choroid. Early frames taken until 2 min (early phase) show the early choroidal circulation (Fig. 10a). Two angiographic phases are however principally relevant and crucial in inflammatory diseases. In the intermediate phase (9 ± 2 min), the ICG molecule is still in the general circulation and this phase shows both the retinal and the choroidal circulations (Fig. 10b). It clearly shows the pattern of choroidal and also retinal vessels in “positive” delineated by ICGA fluorescence within the retinal and choroidal vessels. During the late phase (usually 22 ± 4 min.) choroidal vascular pattern is seen in “negative” (dark), outlined by the background ICGA fluorescence in the choroidal stroma while there is no more fluorescence within the choroidal vessels (Fig. 10c).

#### ICGA standard protocol for posterior uveitis

A standard ICGA protocol to analyse choroiditis has been designed [20–22]. The angiographic procedure comprises three main phases; the early phase up to 2–3 min showing superimposed retinal and choroidal large vessels and incipient exudation of the dye through the choriocapillaris into the choroidal stroma. The intermediate phase at about 10 min shows maximum choroidal stromal background fluorescence and the late phase, that begins at about 22 ± 4 min, shows wash out of the dye from the general circulation with the large choroidal vessels appearing dark against the background stromal fluorescence [21].

#### Schematic interpretation of ICGA in uveitis

When analysing ICGA in posterior inflammatory disorders, crucial differences with FA interpretation have to be borne in mind to correctly analyse the images obtained. During initial circulation, ICG is comparable to fluorescein showing the passage through arteriovenous compartments except that it shows superimposed retinal and choroidal circulations. The difference occurs during recirculation time when ICG is progressively leaking out from the fenestrated choriocapillaris, gradually and physiologically impregnating the whole choroidal thickness.

This process can be altered in two ways that can be associated in the same disease, either there is a decreased fluorescence or an increased fluorescence [21].

##### ICG hypofluorescence

**Fig. 11:**
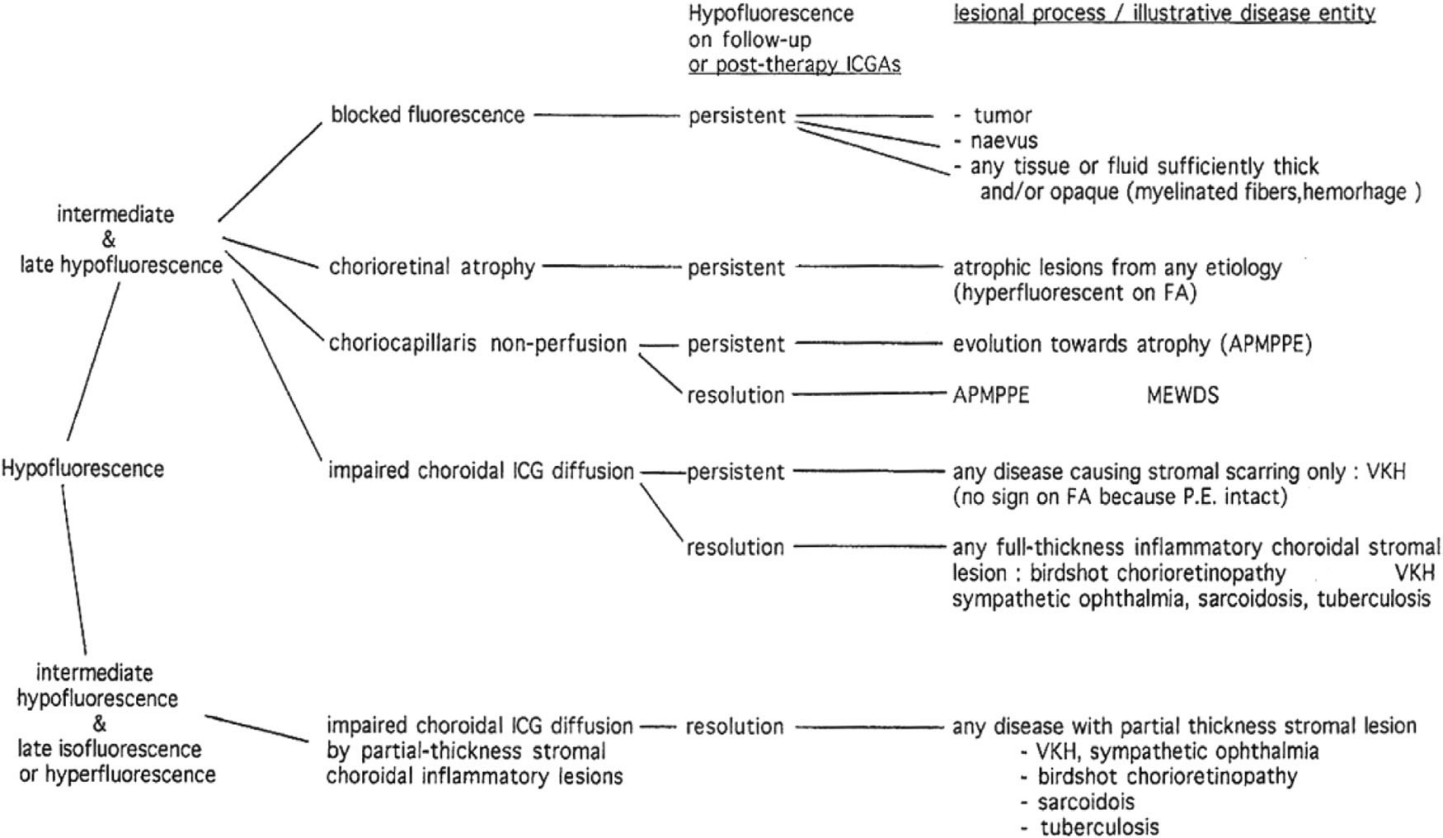
Schematic interpretation of ICGA hypofluorescence (originally published and reproduced from Ophthalmology 1998; 105:432–440).

**Fig. 12:**
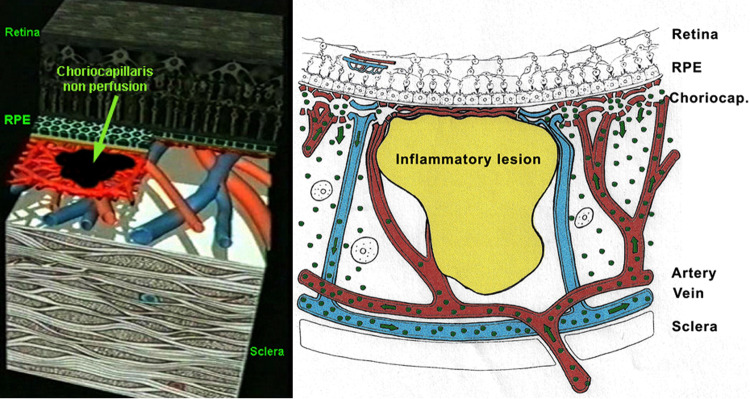
ICG angiographic principles; two mechanisms of hypofluorescence. The physiological fluorescence is impaired by two mechanisms producing hypofluorescent ICGA lesions: (1) choriocapillaris non-perfusion (left picture) occuring in inflammatory choriocapillaropathies and (2) mass effect due to space-occupying lesions such as inflammatory foci here shown as the full-thickness stromal granuloma (right picture) explaining the hypofluorescence seen on ICGA.

The impregnation of the choroidal (Fig. 11) space can be decreased or absent (*hypofluorescence*) either (1) by a decrease of the physiological extrusion of the ICG from the choriocapillaris (non-perfusion or hypoperfusion, confluent geographic aspect) (Figs. 12—left,  13) or (2) by the impairment of the filling of the choroidal tissue by the ICG molecule because of the presence of space-occupying lesions (inflammatory foci, round even in size and regularly distributed) (Figs. 12—right, 14). The latter lesions are hypofluorescent in the intermediate angiographic phase. If they remain hypofluorescent in the late phase this signifies that the inflammatory lesion occupies the whole thickness of the choroidal stroma. When lesions become iso-fluorescent in the late angiographic phase inflammation causes only partial thickness infiltrates. Therefore, in ICGA performed for inflammatory disorders, the main information is obtained less from the analysis of the early circulatory phase than from the analysis of the altered pattern of the filling of the choroidal space.

**Fig. 13:**
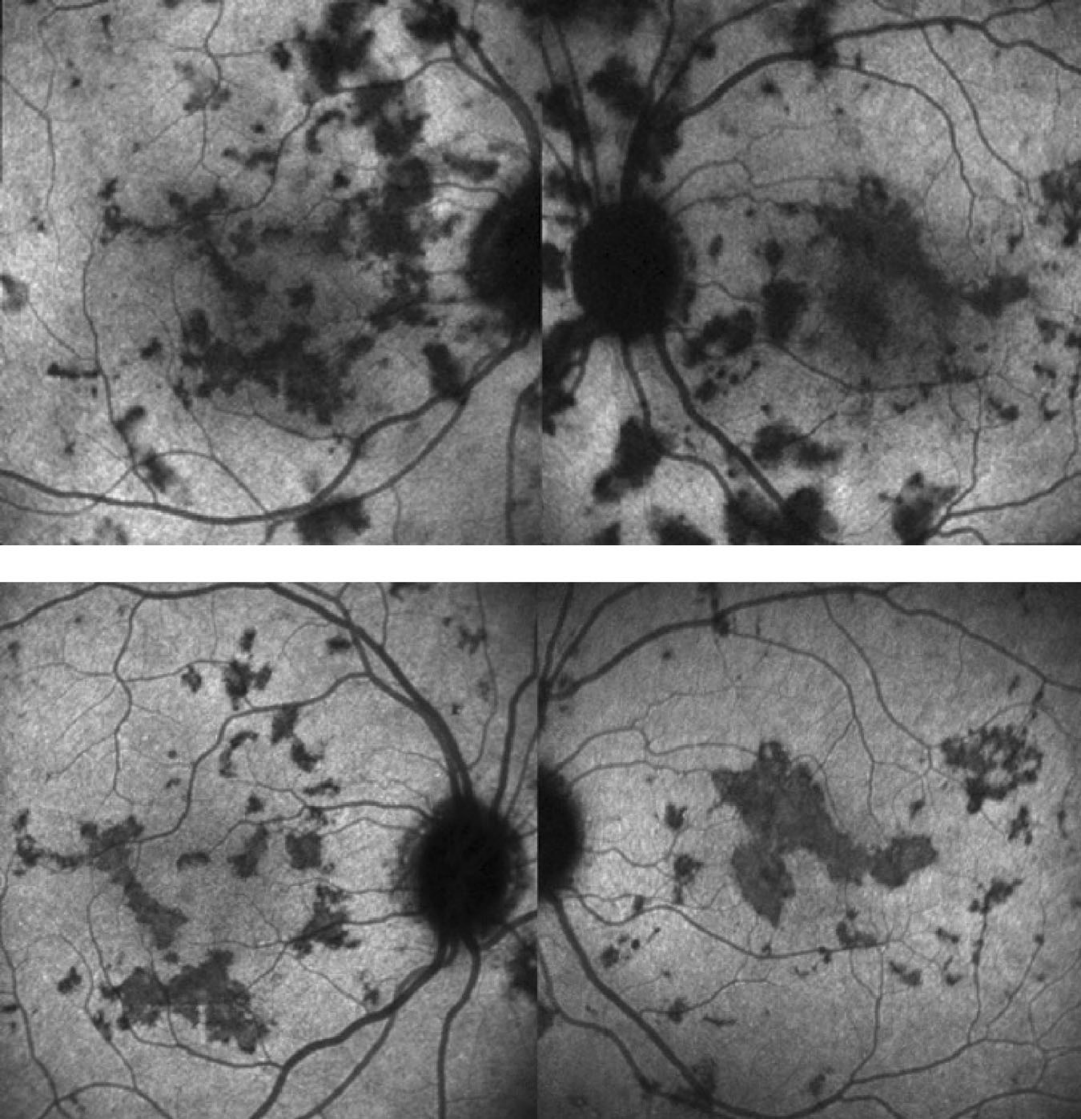
ICGA signs of choriocapillaris non-perfusion. Bilateral areas of patchy or geographic ICGA hypofluorescent areas of variable sizes during the acute phase in a case of APMPPE (top two frames), corresponding to confluent plaques of deep fundal discoloration, often leaving atrophic areas as seen in serpiginous choroiditis in the convalescent phase (bottom two frames).

**Fig. 14:**
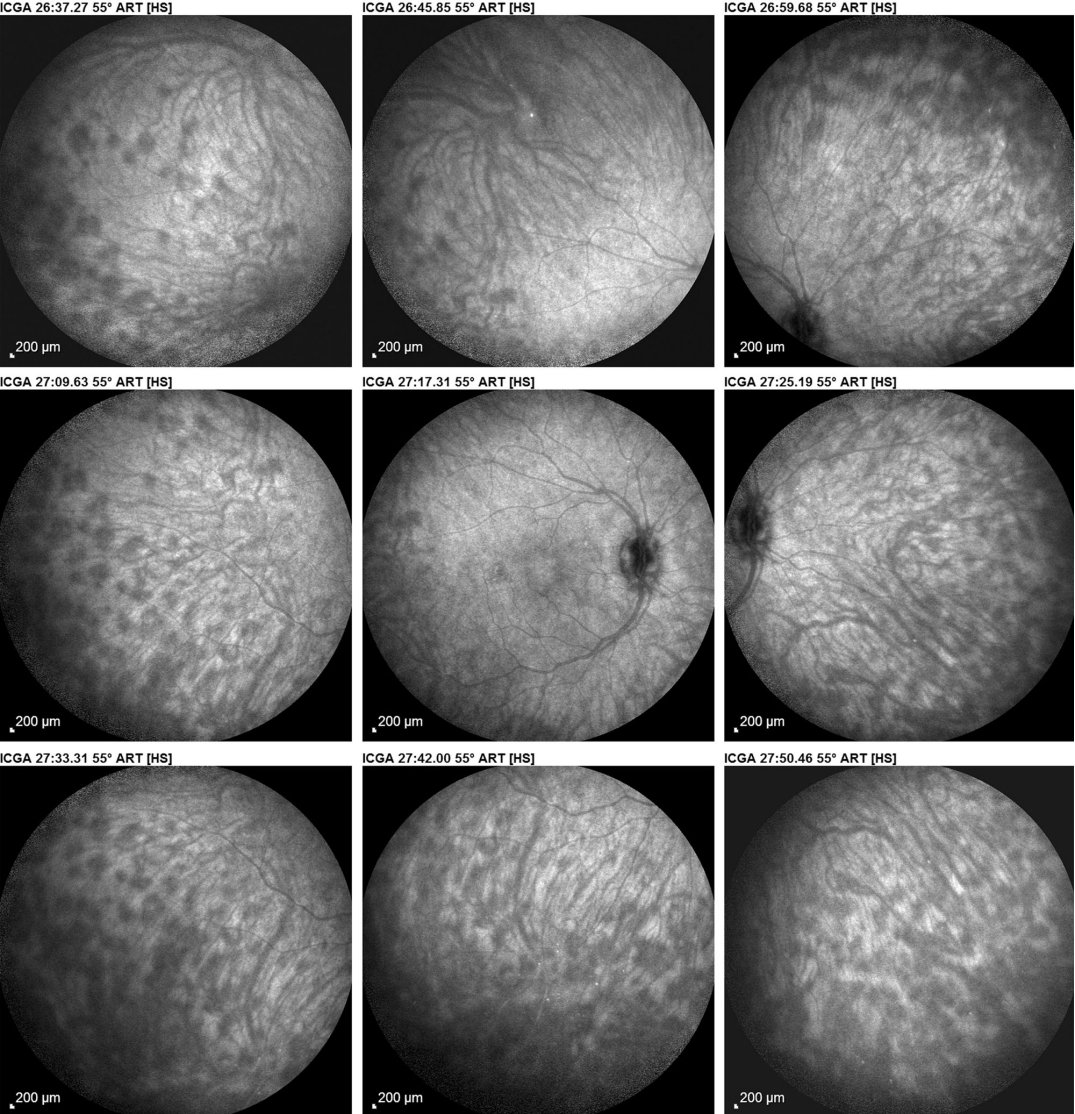
Stromal inflammatory foci/granulomas in Vogt–Koyanagi–Harada disease. Typical regularly distributed hypofluorescent evenly sized dark dots indicating numerous choroidal granulomas.

##### ICG hyperfluorescence

**Fig. 15:**
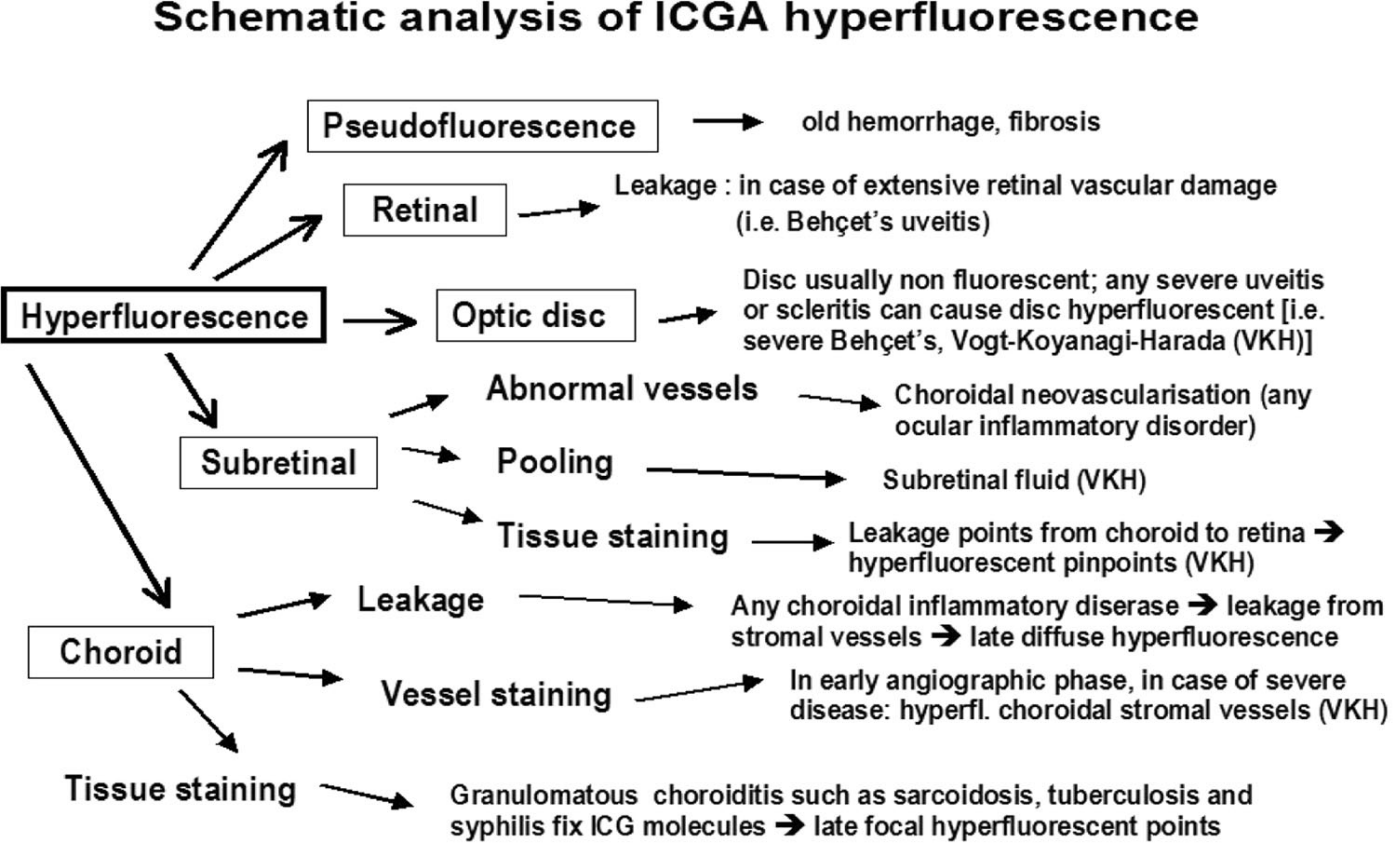
Schematic interpretation of ICGA hyperfluorescence.

**Fig. 16:**
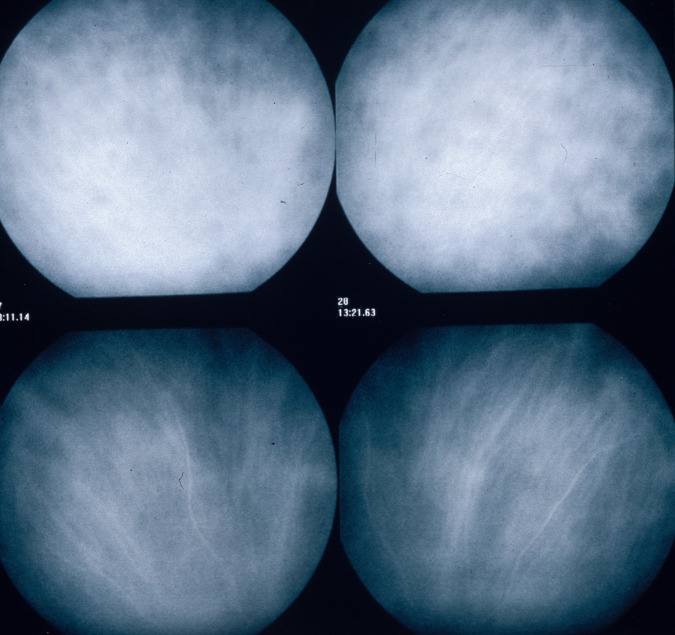
Choroidal stromal vasculitis in Vogt–Koyanagi–Harada disease. Hypofluorescent dark dots surrounded by indistinct fuzzy choroidal vessels (top frames) showing normalisation after only 3 days of pulse intravenous corticosteroid injections (bottom frames).

Impregnation of the choroidal (Fig. 15) space can be enhanced (*hyperfluorescence*) by increased leakage from the larger choroidal vessels which adds to the physiological background fluorescence. The vessels appear fuzzy in the intermediate time frames and extrusion of the dye from large vessels causes late diffuse hyperfluorescence (Fig. 16).

In case of the presence of inflammatory foci in the choroidal stroma, hyperfluorescence is associated with hypofluorescent dark dots due to inflammatory infiltrates.

In most cases, unlike in FA where most pathologies produce hyperfluorescence, the lesions in ICGA are mostly seen in a “negative” dark pattern due to impaired physiological choroïdal fluorescence.

#### Histopathologic correlation with ICGA angiography

The pathologic processes at the origin of the ICGA images have been verified histopathologically for some of the diseases such as the primary stromal choroiditis entities such as VKH disease, sympathetic ophthalmia and HLA-A29 BRC [23, 24], as well as the choroidal lesions of sarcoidosis, while others can still only be hypothesised, still needing ICGA-clinico-pathologic correlations such as the primary inflammatory choriocapillaropathies (PICCPs).

#### Is ICGA still relevant in ocular inflammatory diseases?

ICGA showed occult choroidal lesions not shown by fundoscopy and/or FA in 100% of patients with a well-established diagnosis known to involve the choroid and these findings had an essential impact either on diagnosis or management in 12.3% of these cases, stressing the importance of ICGA for the proper management of most inflammatory processes of the back of the eye [20–22].

#### Use of ICGA applied to uveitis

ICGA is principally useful and has allowed imaging access to the choroidal compartment so far poorly explored. In addition to showing often missed choroidal involvement in every day practice it has allowed to understand choroidal inflammation, classifying choroiditis according to the structure that is preponderantly or initially involved and not simply according to fundus appearance of lesions. At the present stage of our knowledge there seem to be at least two main mechanisms of inflammation touching the choroid, choriocapillaritis [25–27], or stromal choroiditis [28–30]

Two main ICGA derived lesion mechanisms determine the classification of choroiditis:

1. *Choriocapillaris inflammation*
  1.1 Primary inflammatory choriocapillaropathies (primary choriocapillarits entities)

- Multiple Evanescent White Dot Syndrome (MEWDS)/Acute Idiopathic Blind
- Spot Enlargement (AIBSE); (some individuals do not classify it in choriocapillaritis)
- Acute Posterior Multifocal Placoid Pigment Epitheliopathy (APMPPE)/Acute
- Multifocal Ischaemic Choriocapillaropathy (AMIC) [25, 31, 32]
- Idiopathic Multifocal Choroiditis (MFC & Co, PIC) [33]
- Serpiginous Choroiditis
- Rare entities: (Unilateral Acute Idiopathic Maculopathy (UAIM), Acute Zonal Occult Outer Retinopathy (AZOOR)
  1.2 Secondary Inflammatory Choriocapillaropathies
- Acute Syphilitic Posterior Placoid Chorioretinitis (ASPPC)

2. Stromal inflammation (stromal choroiditis) further subdivided into two categories
  2.1 Primary obligatory stromal choroiditiis

- VKH disease [29, 30]
- Sympathetic Ophthalmia [34]
- Birdshot HLA-A29 retinochoroiditis [35, 36]
  2.2. Stromal choroiditis as a random location of a systemic disease
- Sarcoidosis [18]
- Tuberculosis [37]
- Syphilis
- Other infectious choroiditis entities

##### Primary inflammatory choriocapillaropathies/choriocapillaritis entities

This first group of diseases, formerly mostly included in the inadequate term of “white dot syndromes” results from inflammation at the level of the choriocapillaris causing areas of choriocapillaris non-perfusion and its ischaemic consequences both at the level of the choroid but also at the level of the outer retina that depends on the choriocapillaris for oxygen and nutrients [28]. APMPPE is a disease typically illustrating this type of choroidal inflammation [25–27, 31–33].

**Angiographic signs in inflammatory choriocapillaropathies** [25–27, 31–33]: ICG angiographic signs in inflammatory choriocapillaropathies are well determined and have contributed to the recognition of the common mechanism involved and to regroup these entities formerly classified under the term of “white dot syndromes”

The following ICGA signs have to be looked for:

1. The hallmark sign of inflammatory choriocapillaropathy is patchy or geographic ICGA hypofluorescent areas of variable sizes present in the early, intermediate and late angiographic phases but usually more clearly visible on the late frames, indicating choriocapillaris non-perfusion or hypoperfusion (Figs. 12a, 13,  17a, b).

**Fig. 17:**
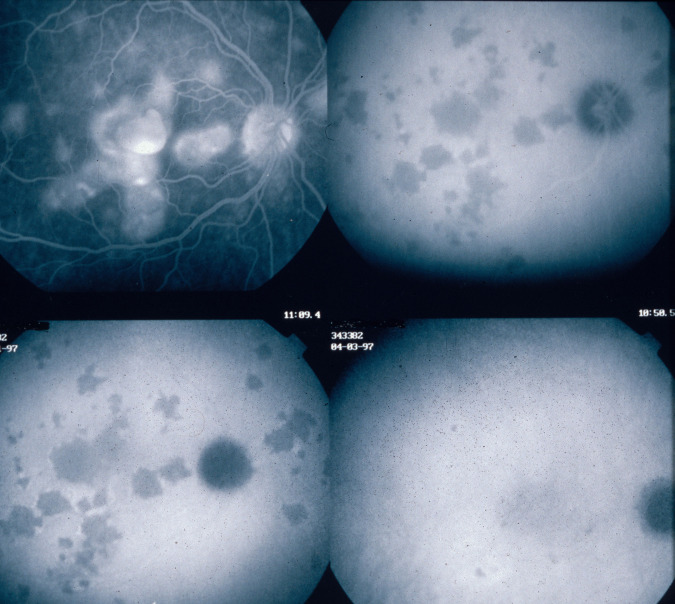

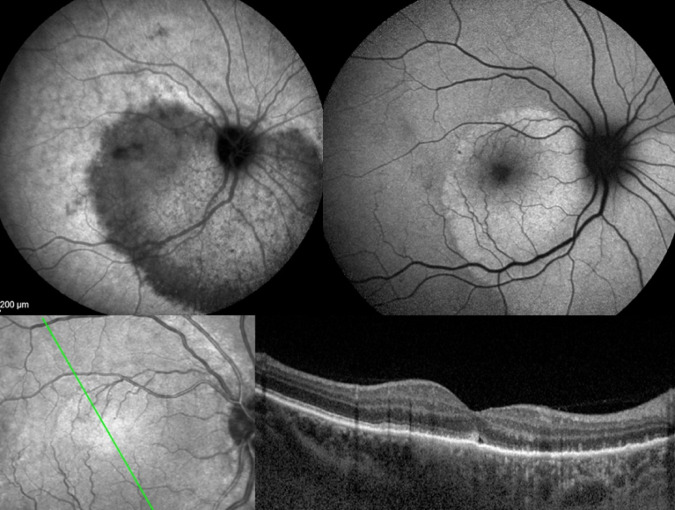
**a** Choriocapillaris non-perfusion in a case of APMPPE/AMIC. Choriocapillaris non-perfusion is shown by patchy geographic areas of hypofluorescence in the intermediate phase of angiography (top right picture) and in the late angiographic phase (bottom left picture) that resolve almost completely in the convalescent stage of disease 2 months later (bottom right picture). The late fluorescein frame on the top left shows hyperfluorescence, corresponding to the ICG areas of choriocapillaris non-perfusion that can only be explained by leakage from the capillaries of the inner retina in response to ischaemic signals from the outer retina. **b** Choriocapillaris non-perfusion in a case of secondary choriocapillaritis. **b** Case Acute Syphilitic Posterior Placoid Chorioretinitis (ASPPC). ICGA (top left) shows choriocapillaris non or hypoperfusion. The area corresponds to FAF hyperautofluorescence (top right) which in turn corresponds to loss of photoreceptor outer segments on OCT (bottom) explaining the FAF hyperautofluorescence. Note also total stromal choroidal thickening on the OCT (bottom image).
2. Complete or partial regression of the ICGA hypofluorescence or absence of regression in the convalescent phase. The areas remaining hypofluorescent in the convalescent phase represent chorioretinal atrophy and correspond to areas of window effect and masking effect on FA.
3. In diseases with a progressing course such as serpiginous choroiditis, ICGA can show diffuse choroidal hyperfluorescence at the edges of the progressing lesions in areas having no translation on fundoscopy or FA (Fig. 18).

**Fig. 18:**
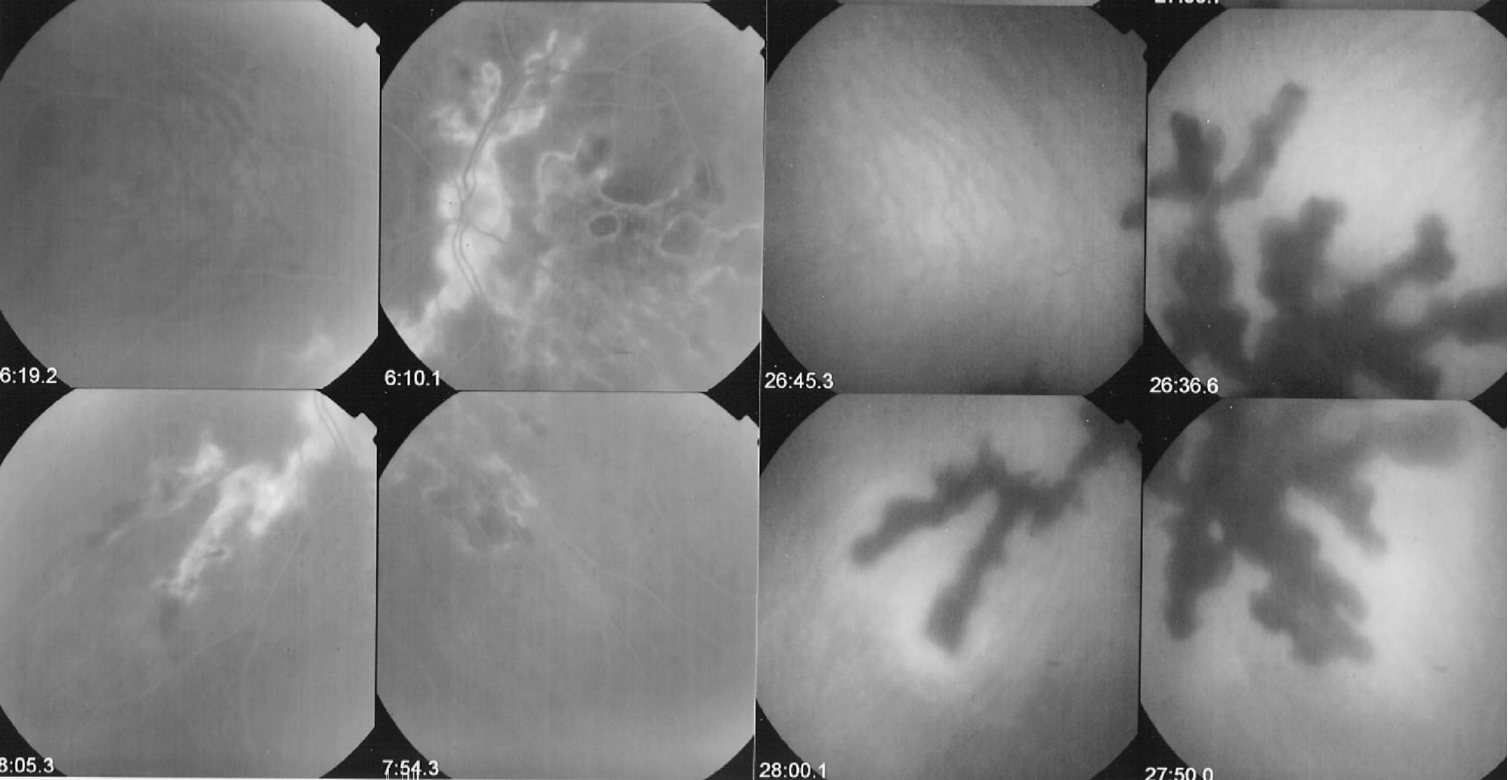
Diffuse perilesional choroidal hyperfluorescence in subclinically progressing serpiginous choroiditis. ICGA (right quartet of pictures) shows many more involved areas than shown by fluorescein angiography (left quartet of pictures) and shows hyperfluorescence around progressing lesions.

Whereas the ICGA signs are quite uniform, the corresponding FA angiographic signs depend on the severity and extension of the choriocapillaris non-perfusion and on the outer retinal damage.

1. On FA there is likewise early hypofluorescence showing the choriocapillaris non-perfusion identified on ICGA.
2. Depending on the severity of the choriocapillaris non-perfusion seen on ICGA, late FA frames either show no hyperfluorescence (for instance in mild MEWDS), discrete patchy late hyperfluorescence seen in MEWDS, or extensive late hyperfluorescence seen in APMPPE (Fig. 17a).
  To understand the genesis of the FA signs in choriocapillaritis it is important to be aware that late FA fluorescence is coming from retinal vessels overlying areas of ischaemic outer retina that present increased permeability in response to the ischemia due to choriocapillaris non-perfusion.
3. In the convalescent phase there is a delayed regression of FA signs (hyperfluorescence and staining) as compared with the regression of ICGA signs.

Any severe inflammation in an adjacent structure to the choriocapillaris (retina or choroidal stroma) can cause inflammation at the level of the choriocapillaris and produce similar angiographic signs. In that situation we speak of secondary inflammatory choriocapillaropathy. The latter term is also used when the triggering agent is known such Treponama Pallidum in acute syphilitic posterior placoid chorioretinitis (Fig. 17b).

##### Stromal choroiditis

**Fig. 19:**
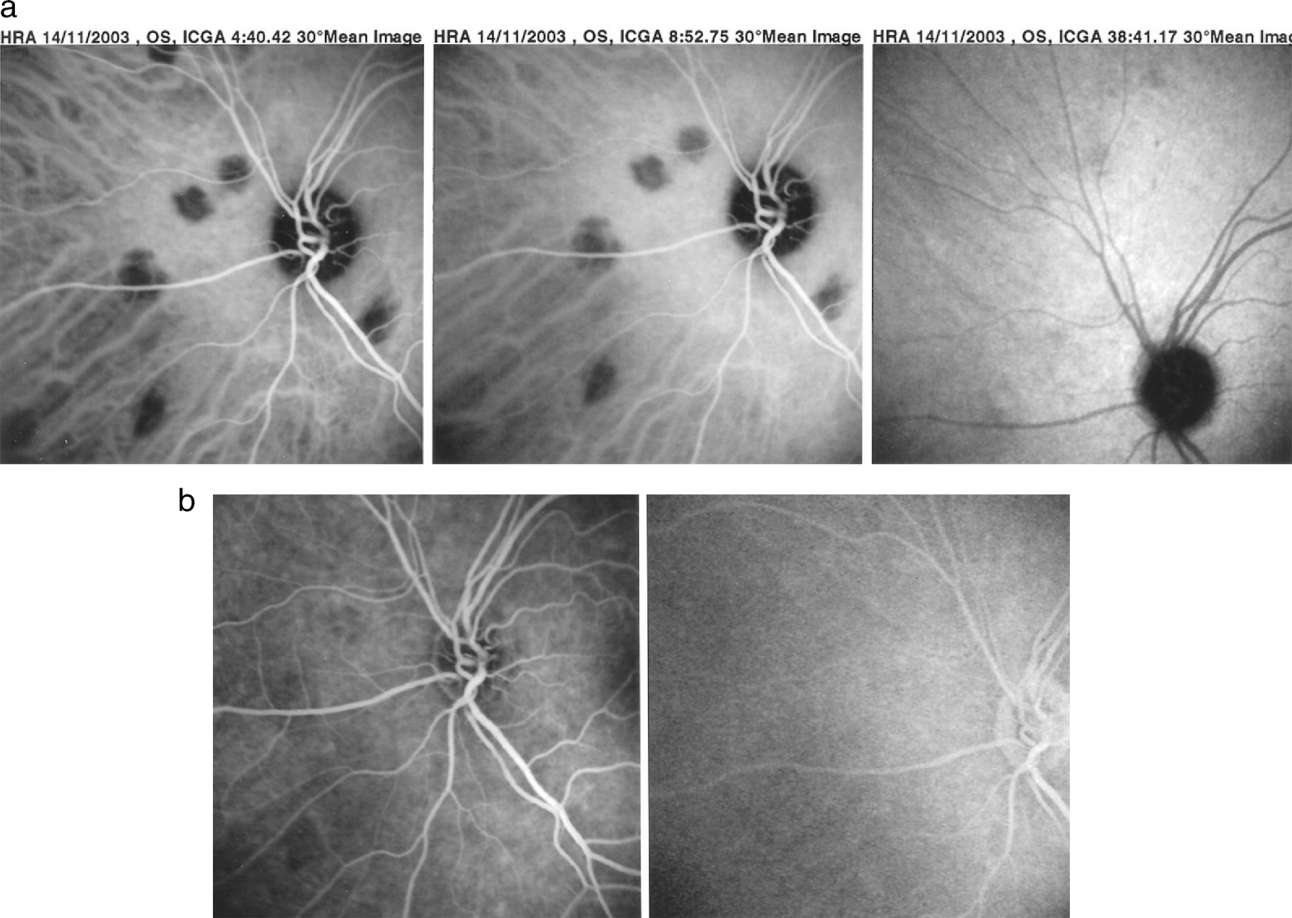
Choroiditis in Sarcoidosis. Unevenly sized randomly distributed lesions (Fig. 19a) well seen in the intermediate ICG angiographic phase (Fig. 19a, middle and left frames) and disappearing in the late phase (right frame), indicating that these presumed granuloma are of partial thickness, not filling the stroma from sclera to choriocapillaris. The lesions are hardly visible on fluorescein angiography (Fig. 19b).

In the second group of diseases, the primary mechanism is the development of inflammatory foci, mostly granulomatous at the level of the stroma appearing hypofluorescent on ICGA, usually associated with inflammation of larger non-fenestrated stromal vessels appearing on ICGA as fuzzy vessels in the intermediate phase followed by diffuse late choroidal hyperfluorescence. VKH disease or HLA-A29 BRC are typical illustrations of this type of choroidal inflammation. For the latter two conditions the lesion process is starting electively within the choroidal stroma and they are therefore called primary stromal choroiditis. In other cases such as sarcoidosis chorioretinitis the choroidal stroma is just one chance localisation of a systemic process (an innocent bystander) and are therefore called secondary stromal choroiditis entities. In contrast to primary stromal choroiditis where the distribution of hypofluorescent dark dots (HDDs) is uniform and their size is regular, in secondary stromal choroiditis HDDs have a random distribution (Fig. 19). Although the mechanism of primary stromal choroiditis is completely different from choriocapillaritis, these conditions have also been included inadequately by some authors in the “white dot syndromes”.

#### Additional ICGA signs

Apart from the signs that allow to explore choroidal inflammation, whether at the level of the choriocapillaris or at the level of the choroidal stroma, additional ICGA signs have been reported.

The optic disc is usually non fluorescent in ICGA. In case of severe inflammatory disease disc hyperfluorescence is a characteristic finding usually present in all types of hyperacute diseases such as initial attacks of Behçet’s uveitis and VKH disease, representing also a parameter to monitor the effect of therapeutic intervention. In a group of VKH patients, it was shown to regress within 1 month after introduction of systemic therapy [29].

### ICGA impact in the practical care of the uveitis patient

#### Precise assessment of choroidal inflammatory involvement

The choroid is the starting point of the inflammation in many diseases including MEWDS, APMPPE, multifocal choroiditis, serpiginous choroiditis, VKH, sympathetic ophthalmia and HLA-A29 BRC and is participating in the inflammatory process in many other diseases such as sarcoidosis, tuberculosis, syphilis, toxoplasmosis, posterior scleritis and many more which have extensively been analysed by ICGA [37–40]. A reliable inventory of fundus inflammatory involvement can therefore only be performed with the help of ICGA. It will contribute essentially to the assessment of disease extension in those conditions involving the choroid.

**Fig. 20:**
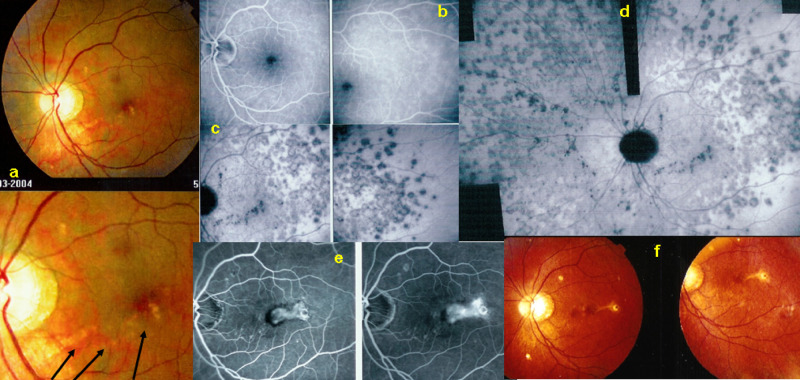
Multifocal choroiditis (the jellyfish effect or constellation). Fundus pictures show minimal signs limited to faint depigmentation (**a**, black arrows) and FA shows absolutely no abnormalities (**b**), whereas ICGA shows extensive choriocapillaris involvement with large geographic areas of non-perfusion (**c**, **d**) which we now know to be a risk factor for CNV, which the patient developed 1 year later as seen on FA (**e**) and fundus images (**f**).

**Fig. 21:**
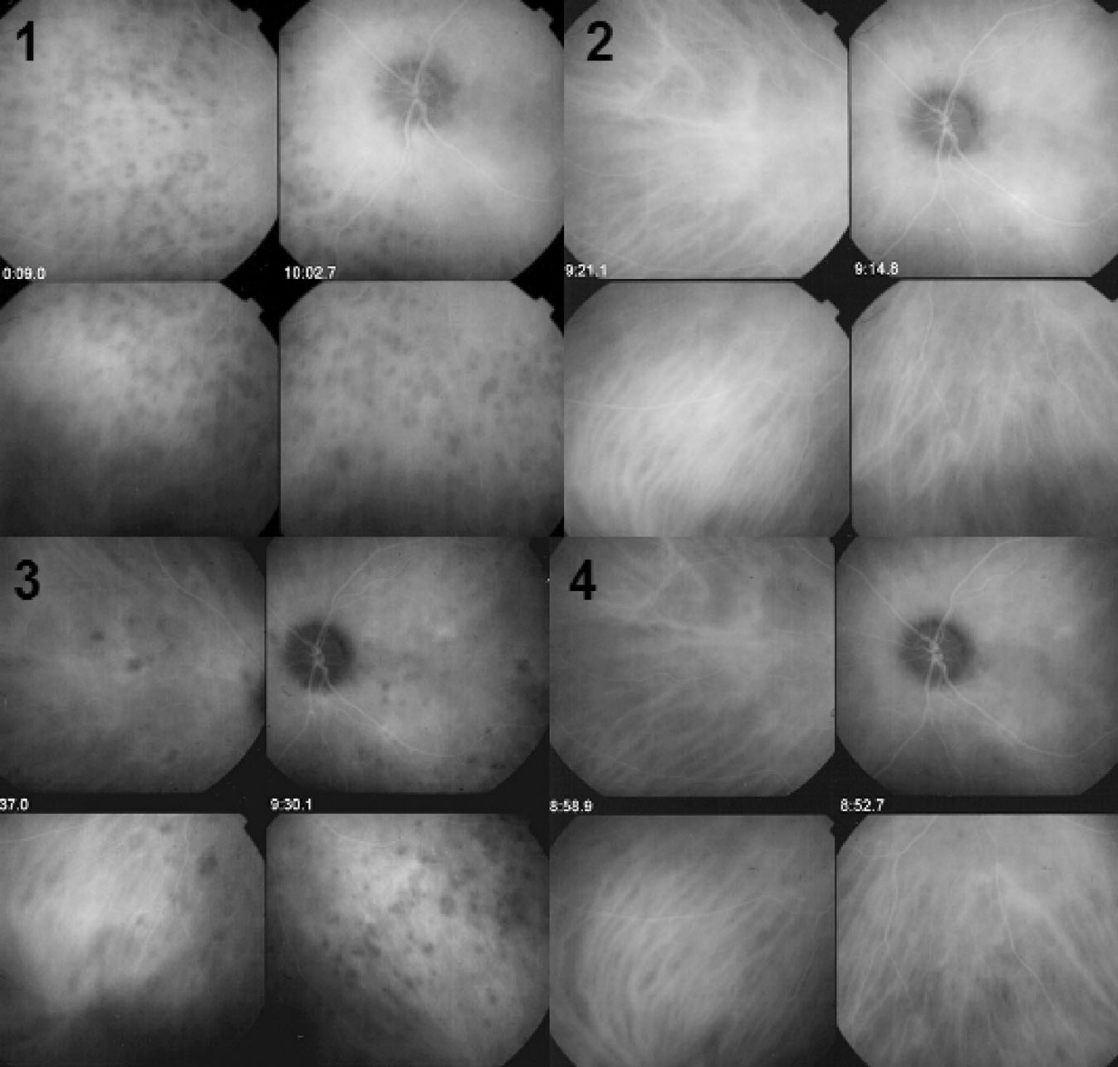
ICGA guided treatment of VKH disease. VKH at presentation (1 = top left): numerous hypofluorescent dark dots (HDDs) indicating granulomatous foci in the choroid as well as fuzzy choroidal vessels indicating choroidal vasculitis. Following intravenous pulse steroid therapy followed by high dose oral steroids HDDs disappear completely and choroidal vessels regain a normal pattern (2 = top right). Upon tapering there is recrudescence of subclinical choroidal disease (3 = bottom left), that prompts re-increase of oral steroid therapy and introduction of azathioprine. After slow tapering of steroid therapy first followed by tapering of azathioprine over 2 years the patient remains recurrence free (4 = bottom right).

In some diseases such as MEWDS only choroidal signs can be present and in others the clinically apparent part of the disease such as in multifocal choroiditis is only the peak of the iceberg (jellyfish effect) and the preponderant part of the disease can only be followed by ICGA (Fig. 20). In diseases originating from the choroid such as VKH disease the only way to detect subclinical disease is to perform ICGA (Fig. 21).

#### Diagnostic contribution of ICGA

**Fig. 22:**
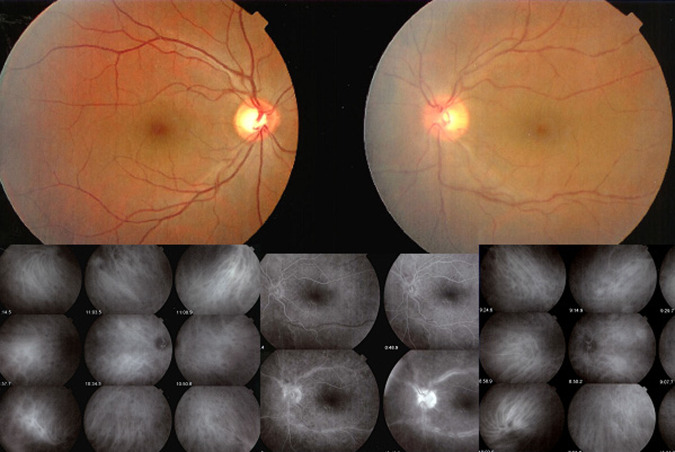
The essential role of ICGA for early diagnosis of birdshot HLA-A29 retinochoroiditis. Patient in his mid-forties presenting with blurred vision OS due to the presence of vitritis (top right). FA shows retinal vasculitis OS of large veins and small vessels (bottom middle). The right eye seems not affected (top left). However, ICGA shows numerous hypofluorescent dark dots on the left (bottom right) but also on the right (bottom left), typical for birdshot HLA-A29 retinochoroiditis.

**Fig. 23:**
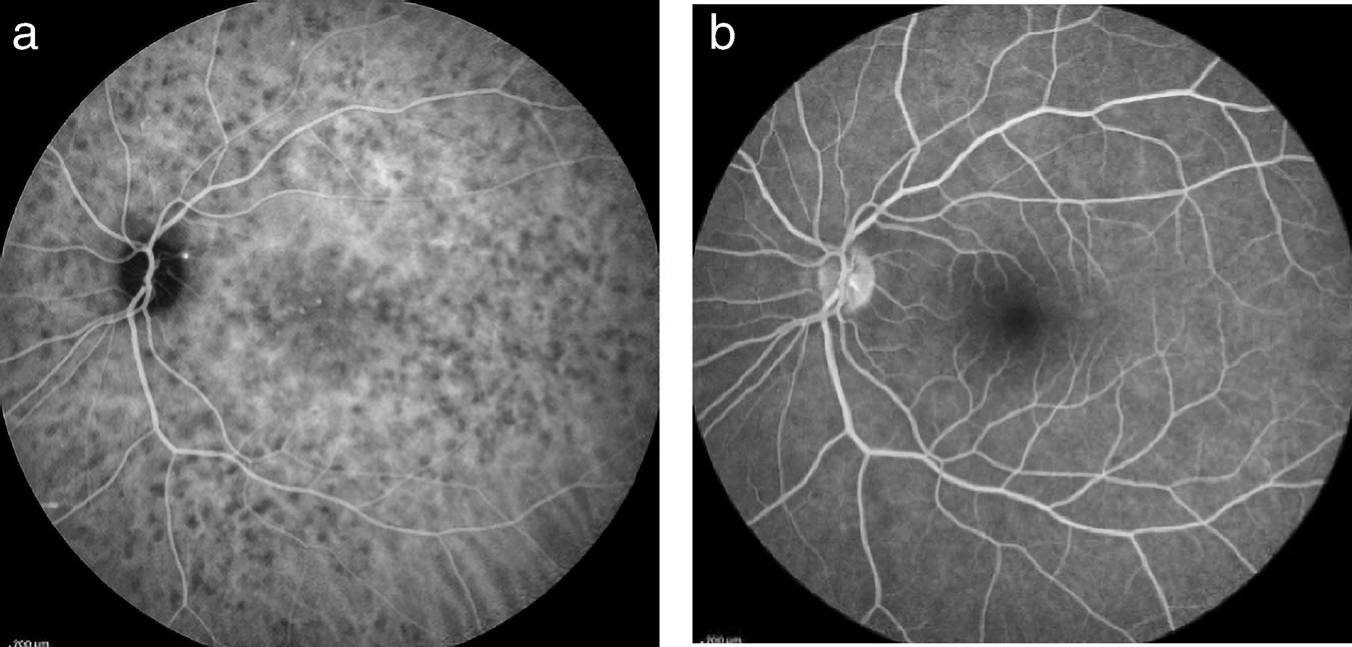
**a** (left) and **b** (right). The essential role of ICGA for early diagnosis of VKH disease. ICGA intermediate phase shows numerous hypofluorescent dark dots (HDDs) (left frame) while FA looks normal.

Unlike for FA, it is not rare that ICGA gives the essential contribution that leads to the diagnosis. This can be the case for patients with MEWDS that have minimal translation in fundus signs or FA signs. Early HLA-A29 BRC can only be diagnosed using ICGA as the early lesions are HDDs indicating subclinical granulomas that can only be detected by ICGA. These ICGA lesions often precede the typical oval shaped depigmented “birdshot” lesions by several months or even years. (Fig. 22) The same is true for VKH disease (Fig. 23).

#### ICGA monitoring of disease evolution and response to therapy

As choroidal lesions can only be detected by ICGA, it is obviously also the recommended modality to monitor the evolution and to evaluate the impact of treatment on choroidal inflammatory processes. In case of VKH disease, clinical disease, meaning inflammation involving extrachoroidal structures accessible to fundus observation, to OCT and FA, can be followed by classical means. However once clinical disease is under control, it has been shown that subclinical disease is ongoing resulting in almost 100% of cases in sunset-glow-fundus (SGF) the witness of ongoing disease destroying choroidal pigment. It was recently shown that ICGA guided treatment of VKH could avoid evolution towards SGF when treating also subclinical disease shown by ICGA (Fig. 21).

### Conclusion: the place of ICGA in multimodal imaging of posterior uveitis

**Fig. 24:**
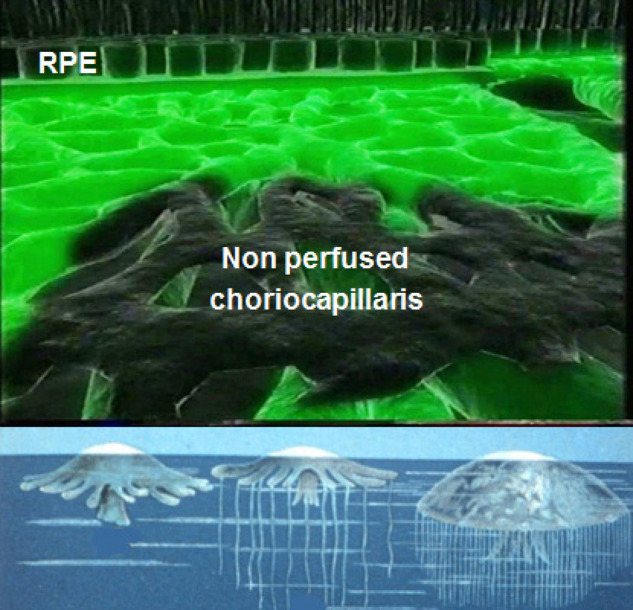
Jellyfish effect: cartoon on the mechanism involved in choriocapillaritis and its ICGA imaging. The top image shows an area of inflammatory non-perfusion of the choriocapillaris. The bottom image is an analogy of the type of lesion process involved in choriocapillaritis. Similar to jellyfish in the seas, lingering unseen under the surface of the water, damage to the choriocapillaris occurs undetected below the retinal pigment epithelium, unless ICGA is performed.

**Fig. 25:**
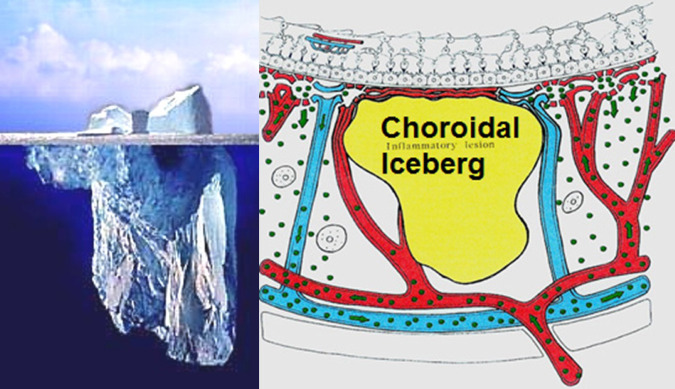
Cartoon on the mechanism involved in stromal choroiditis and its ICGA imaging. The right image shows an inflammatory lesion filling the choroidal stroma. The left image is an analogy of the type of lesion process involved in stromal choroiditis. Similar to an iceberg where the bulk of ice is unseen under the surface of the water, the major or whole part of the lesion in stromal choroiditis is undetected below the retinal pigment epithelium, unless ICGA is performed.

FA is performed routinely in inflammatory ocular diseases, which is probably justified in order to make a good evaluation of inflammation and to use it as a follow-up parameter for superficial fundus structures. Except for the evaluation of retinal vessels it mostly does not add essential information to clinical examination and OCT. In contrast, ICGA often furnishes additional information otherwise undetected on the choroidal compartment. Several choroidal diseases such as PICCPs (MEWDS, multifocal choroiditis, APMPPE and others) present with the “jellyfish effect or constellation” meaning presence of lesions of the choriocapillaris below the RPE with no or minimal signs seen on funduscopy or FA with nevertheless extensive choroidal involvement (Fig. 24). Similarly in stromal choroiditis entities such as VKH or birdshot, choroidal lesions would be mostly undetected unless ICGA were performed. In this case, the analogy of the “iceberg effect or constellation” best characterises the lesion process of bulky occult foci located in the choroidal stroma (Fig. 25).

Therefore, except for conditions such as pars planitis or Behçet’s disease where choroiditis is absent or insignificant, dual FA and ICGA should be performed for the assessment of posterior uveitis if angiographic analysis is deemed necessary as choroiditis cannot be excluded “a priory”. A dual FA/ICGA angiographic scoring method has recently been proposed for that very purpose.

### Practical summary

Uveitis can be considered to have developed into an exact “clinical science”, as investigational procedures allow increasingly precise appraisal of lesions in most ocular compartments. ICGA is the method of choice to explore choroidal inflammation. It is giving essential information no other method can furnish to assess and follow choroiditis. It is the most sensitive and reactive means to monitor therapeutic intervention and in that regard is not matched by new investigations such as enhanced depth imaging (EDI-OCT) measurement of choroidal thickness. Therefore, management of posterior uveitis where choroid is involved cannot be meaningfully performed without ICGA.

## Dual FA/ICGA angiographic scoring system

A semiquantitative angiographic scoring system was developed by the Angiography Scoring for Uveitis Working Group (ASUWOG) in 2010 [12] and has proven interobserver reliability for both conventional and wide-field angiography [13, 41]. The total maximum FA score is 40, including optic disc hyperfluorescence (score of 3), macular oedema (score of 4), retinal vascular staining/leakage (score of 7), capillary leakage (score of 10), retinal capillary non-perfusion (score of 6), neovascularization of the disc and elsewhere (score of 4), pinpoint leaks (score of 2), and retinal staining/pooling (score of 4). The ICGA signs include four categories: early stromal vessel hyperfluorescence (score of 3), fuzzy choroidal vessels (score of 6), dark dots excluding atrophy (score of 8), and optic disc hyperfluorescence (score of 3); thus, the total maximum ICGA score is 20. The ICGA score is multiplied by a coefficient of 2 so that the inflammation in the retina versus the choroid can be directly compared between FA and ICGA.

Angiography is based on the deviation from what is considered as normal fluorescence towards hyper or hypofluorescence. This is the case for both retinal and choroidal delta-fluorescence. The interpretation of inflammatory signs in angiography is based on this principle of hyper and/or hypofluorescence.

Based on this classical phenomenon, the ASUWOG group defined inflammation score points for both retinal and choroidal delta-fluorescence. There are twice as many structures that can cause delta-fluorescence in the retina when compared with the choroid, which has less capacity to express inflammation score points based on delta-fluorescence. Therefore, its capacity to express inflammation score points is going to be artificially lower and had to be adjusted so that the inflammation score points generated by hyper/hypofluorescence could be compared between the retina and the choroid. The advantage of such a system is that choroidal and retinal inflammation can be comparatively evaluated, as inflammation points based on delta-fluorescence were the common denominator, a phenomenon occurring similarly in both structures [42]. These principles resulted in the angiography scoring system of ASUWOG, a collaborative work including 16 colleagues from 9 countries experienced in angiography for inflammation. This system can be seen as one of significant steps towards precise evaluation of posterior uveitis.

The dual FA/ICGA scoring system has been used for comparison of retinal and choroidal inflammation [4, 43–46] as well as to document treatment response in each compartment in a variety of uveitic entities [47–50].

The FA scoring system has also been used separately in studies of Behçet uveitis which primarily involves the retinal vasculature [51–54].

## Conclusion

Although, in present times, there is a continued trend towards non-invasive imaging techniques, ocular angiography still represents the mainstay of routine investigation of posterior uveitis when it is severe enough to warrant energic investigation. OCT to investigate the retina, or the choroid by EDI-OCT, have been valuable non-invasive additions, among others, for the appraisal of posterior uveitis. Their advantage is that examinations can be easily repeated and are especially useful for follow-up purposes. At present, however, to detect inflammatory macular oedema or retinal vasculitis, FA is still more sensitive than OCT [55]. Similarly ICGA is still more accurate than EDI-OCT to detect and follow choroiditis, especially because it gives global information on the whole fundus whereas EDI-OCT only yields information on the posterior pole [47, 56]. The combination of angiographic and non-invasive methods represents, without any doubt, a substantial evolution towards precise evaluation of posterior uveitis that should replace obsolete methods still in use today [57].
